# Differences in Force-Velocity Profiles During Countermovement Jump and Flywheel Squats and Associations With a Different Change of Direction Tests in Elite Karatekas

**DOI:** 10.3389/fphys.2022.828394

**Published:** 2022-06-21

**Authors:** Darjan Smajla, Darjan Spudić, Žiga Kozinc, Nejc Šarabon

**Affiliations:** ^1^ Faculty of Health Sciences, University of Primorska, Izola, Slovenia; ^2^ Human Health Department, InnoRenew CoE, Izola, Slovenia; ^3^ Faculty of Sports, University of Ljubljana, Ljubljana, Slovenia; ^4^ S2P, Science to Practice, Ltd., Laboratory for Motor Control and Motor Behavior, Ljubljana, Slovenia

**Keywords:** load, combat, kumite, performance, power

## Abstract

The force-velocity (F-v) relationship has been proposed as a biomechanical characteristic to comprehensively evaluate neuromuscular capabilities within different tasks such as vertical jumping, sprinting and bench pressing. F-v relationship during flywheel (FW) squats was already validated, however, it was never compared to F-v profile of vertical jumps or associated with change of direction (CoD) performance. The aims of our study were (1) to compare F-v profiles measured during counter movement jumps (CMJs) and FW squats, (2) to determine correlations of F-v mechanical capacities with different CoD tests, (3) to investigate the portion of explained variance in CoD tests with the F-v outcome measures. A cross-sectional study was conducted on 39 elite karatekas. They performed CMJs and FW squats using progressive loads to calculate F-v profile outcome variables and different CoD tests (CoD at 90°, CoD at 180°, *t*-test, short karate specific test (KST) and long KST). Our results showed significantly higher values in all F-v outcome variables (F_0_—theoretical maximal force, V_0_—maximal unloaded velocity, P_max_—maximal power output, F-v_slope_—the slope of F-v relationship) calculated from CMJs compared to FW squats (all *p* < 0.01). Significant positive moderate correlations between the tasks were found for F_0_ and P_max_ (r = 0.323–0.378, *p* = 0.018–0.045). In comparison to F-v outcome variables obtained in FW squats, higher correlations were found between F-v outcome variables calculated from CMJs and CoD tests. The only significant correlation in F-v outcome variables calculated from FW squats was found between P_max_ and short KST time. For all CoD tests, only one F-v predictor was included; more specifically—CMJ-F_0_ for CoD 90°, CoD 180° and *t*-test, and FW-P_max_ for short KST performance. To conclude, our results showed that F-v relationship between CMJs and FW squats differed significantly and cannot be used interchangeably for F-v profiling. Moreover, we confirmed that high force and power production is important for the successful performance of general and karate specific CoD tasks.

## Introduction

Modern karate tournaments under World Karate Federations consist of two disciplines, the kumite and kata. In a kumite fight, two participants perform different attacking and defensive techniques against each other (Imamura et al., 2002). Kumite popularity has been growing, especially after the official representation of karate kumite at the 2020 Olympic Games in Tokyo. Regardless of its growing popularity, there is a lack of attention to all aspects of this sport in scientific literature.

The duration of a kumite match is 3 min of combat time for men and women, and it is performed on an 8 × 8 m mat. During the match, forward, backward, sidestepping and hopping movements are performed at different intensities. Most of the movements are performed at low intensity, while shorter sequences of attacking and defending techniques are performed at maximum intensity (Beneke et al., 2004). Fast leg movement in karate guard during explosive actions, including kicking and punching, requires high lower-limb power production (Chaabène et al., 2012; Loturco et al., 2014). It was identified that power output, change of direction (CoD) ability and speed are the most important determinants of technical and fighting efficiency of karate athletes (Blažević et al., 2006). Power output, CoD ability and speed are important in a wide variety of sports such as soccer (Barnes et al., 2014), basketball (García et al., 2020) or tennis (Fernandez et al., 2009). While these physical determinants have been extensively studied in the aforementioned sports, less research has focused on karate, especially in terms of association to sport-specific performance. Previous studies found that more powerful and stronger athletes usually outperform weaker counterparts in sprinting, jumping and CoD tasks (Nimphius et al., 2010; Spiteri et al., 2015; Pereira et al., 2018). However, some studies did not find significant associations between strength or power and CoD performance (Spiteri et al., 2014; Loturco et al., 2019).

Most of the previously mentioned studies assessed strength and power with one repetition maximum, jump height and sprint time. It was reported that such assessments do not provide a comprehensive insight into neuromuscular capabilities related to push-off performance, as compared to force-velocity (F-v) profiling (Morin and Samozino, 2016). The F-v relationship has been proposed as a testing procedure to comprehensively evaluate neuromuscular capabilities within vertical jumping, sprinting (Morin and Samozino, 2016; Jiménez-Reyes et al., 2017) and bench press throwing (Baena-Raya et al., 2021e) tasks. The F-v relationship represents the athlete’s maximal capacity to produce theoretical maximal force (F_0_), maximal unloaded velocity (V_0_) and maximal power output (P_max_). In addition, the slope of the F-v relationship (F-v_slope_) reflects the balance between force and velocity capacities (Samozino et al., 2012). These mechanical properties have been studied in relation to CoD performance to provide a better understanding of the physical capacities that determine CoD ability. In general, F-v profile outcome measures can discriminate between high and low-level athletes and present significant associations with different sports performance outcomes (Baena-Raya et al., 2021a) It has been shown that horizontal F-v profile (sprints) shows larger associations with CoD performance compared to vertical F-v profile (jumps) in tennis, basketball and soccer players (Baena-Raya et al., 2021d). Moreover, horizontal F-v outcome measures as P_max_ and V_0_ seem to be determinant factors for successful CoD performance (Baena-Raya et al., 2021b). On the other hand, F_0_ during vertical jumping and bench press throwing was associated with sport-specific performance indicators, such as spike and serve ball speed in volleyball (Baena-Raya et al., 2021e). Additionally, some studies showed that P_max_ capacity during cycling (Zivkovic et al., 2017), jumping and sprinting (Marcote-Pequeño et al., 2019) were inter-related, while F_0_ and V_0_ seem to be more task-specific. Based on biomechanical demands of the karate that include short rapid active and reactive full-body movements (Chaabene et al., 2019) F-v profiling using short and explosive actions (e.g. vertical jumps) seems to be a more appropriate testing procedure, compared to sprinting.

Specific test batteries for the most popular sports and their associations with mechanical properties are well established (Baena-Raya et al., 2021d), however, there is a lack of karate-specific tests and protocols (Smajla et al., 2021a). The knowledge of associations between test scores and sport-specific performance could help coaches and karatekas in designing and adjusting the training regime. Rapid and specific movements during karate guarding impose significant demands on lower leg muscles, so the neuromuscular properties of these muscles could be associated to karate performance (Sbriccoli et al., 2010). There is a lack of evidence about F-v profiles and its associations with sport-specific performance in karate, as previous studies have been mainly limited to assessing aerobic and anaerobic metabolism during different karate actions (Nunan, 2006; Chaabene et al., 2012; Tabben et al., 2014).

Producing high power in sport-specific situations maximizes the effectiveness of the movement. For example, jumping and sprinting performance is highly dependent on peak power production of the lower extremities (Jaric and Markovic, 2013) and, in more detail, the optimal balance between force and velocity capabilities contributing to a common peak power production (Samozino et al., 2014). While it has been shown that flywheel (FW) inertial squat training can be an effective tool to improve muscular power (Petré et al., 2018), and based on the findings supporting the link between power production and dynamic athletic performance (Cormie et al., 2011), it is logical that performance in sports activities can be improved after FW inertial resistance training (Raya-González et al., 2021). Vertical F-v profiling using loaded jumps and its associations with specific sport movement have already been investigated in volleyball (Baena-Raya et al., 2021e), soccer (Loturco et al., 2019; Marcote-Pequeño et al., 2019) and athletics (Baena-Raya et al., 2021c; Baena-Raya et al., 2021d). On the other hand, there is a lack of studies about F-v profile during FW squats and its associations with sport-specific performance. FW devices are used as a training tool in resistance training programs and it has been shown that FW training improves jumping and CoD ability, as well as linear speed and reactive strength (De Hoyo et al., 2015; Tous-Fajardo et al., 2016; Coratella et al., 2019). However, even though the calculation of the F-v profile during FW squats using four different progressive inertial loads has been validated (Spudić et al., 2020), there is no studies investigating the differences between F-v profiles obtained using FW squats or vertical jumps and their associations with sport-specific tests. Specifically, the relevance of the F-v profile in FW squats to karate performance and the potential benefit compared to the F-v profile of vertical jumps to karate performance is unknown. A fast preparatory countermovement and subsequent concentric action are important for karate performance (Beneke et al., 2004). Similarly, FW squat performance depends on the ability of athletes to make a quick transition from the eccentric to the concentric portion of the squat and therefore could be a representative measure of karate-specific movement pattern. This could be mainly due to the use of a harness, where only part of the additional load is applied to the trunk extensors (Spudić et al., 2021a) which are commonly a weak point when lifting heavy weights from a barbell squat.

In a study performed by Jiménez-Reyes et al. (2018) authors reported that P_max_ scores obtained with jumping and sprinting F-v profiling are moderately related, while this was not the case with F_0_ and V_0_ (Jiménez-Reyes et al., 2018). Another recent study (Junge et al., 2021) that included sprint, jump and hip thrust tasks corroborated this finding, while Zivkovic et al. (2017) showed some between-tasks associations for F_0_ and V_0_ as well. In the case of consistency of F-v relationships across tasks, performing only one task for assessment purposes would be sufficient. However, as explained above, the literature to date suggests limited associations between F_0_ and V_0_ across different tasks. While P_max_ is perhaps a more universal characteristic, F_0_ and V_0_ appear to be highly task specific. For example, F-v outcome measures during CMJ are significantly higher compared to F-v outcome measures during squat jumps (Jiménez-Reyes et al., 2014). To our knowledge, there are no studies that compared F-v profiles calculated from CMJ and FW squats. In general, CMJ and FW squats are very similar in terms of kinematics, thus, higher associations between F-v outcome measurescould be expected than previously observed between jump and sprint or jump and hip thrust tasks. On the other hand, there are some differences between CMJ execution (full leg extension and maximal acceleration during the jump) and FW squats (reduction of acceleration before the full leg extension) in the execution and tempo of the motor tasks (Spudić et al., 2021a).

Therefore, the first aim of our study was to compare F-v profiles measured during vertical jumps and FW squats and investigate their associations. We hypothesized that F-v profile outcome measures (F_0_, V_0_, P_max_) will be different between vertical jumps and FW squat jumps, while the associations between analogue outcome measures of vertical jumps and FW squats will be moderate to high. The second aim of our study was to evaluate associations between F-v outcome measures and different CoD tests (CoD 90°, CoD 180° and karate specific test (KST)). We hypothesized that significant associations will be found between CMJs and FW squats F-v outcomes and CoD tests times, while higher associations will be found in CMJ F-v outcome measures. The third aim of our study was to investigate the portion of explained variance in CoD tests with the F-v outcome measures (F_0_, V_0_ and P_max_) calculated from CMJ and FW squats. We hypothesized that F-v outcome measures will explain at least 20% of the variance in CoD 90° and at least 30% of the variance in CoD 180° and KST.

## Materials and Methods

### Participants

Thirty-nine international level karatekas participated in this cross-sectional study (Table 1). The measurements were performed in the Austrian national karate sports complex from 9 to 11^th^ September 2020. The inclusion criteria for the study were minimally two training sessions per week in last 2 years including experience in resistance training and at least 5 years of training history in karate. In case of any lower limb injuries, neurological disorders and low back pain in the past 6 months, the participants were excluded from the study. The leg preference for CoD tests was determined by asking participants: “Which leg do you prefer when performing unilateral jumping movements”. The preferred guard was determined by the question: “Which is your preferred front leg during a kumite match?”. All the participants (or their parents/guardians - in case participants were under the age of 18) were informed about the testing procedures and provided informed consent. All participants were instructed to avoid very intense physical activities at least 48 h prior to testing. Slovenian Medical Ethics Committee approved (no. 0120-99/2018/5) the study which was conducted according to the Declaration of Helsinki guidelines.

**TABLE 1:** Characteristics of participants.

Group	N	Age (years)	Body height (cm)	Body mass (kg)	BMI (kg/m^2^)	Training history (years)	Number of training sessions (n/week)
Male	22	19.5 ± 3.9	178.9 ± 4.7	71.4 ± 10.5	22.7 ± 2.8	11.1 ± 4.9	5,6 ± 3.1
Female	17	19.1 ± 4.4	167.5 ± 6.9	58.8 ± 6.0	20.7 ± 1.5	11.9 ± 7.0	6.4 ± 1.7
All	39	19.3 ± 4.1	173.2 ± 8.2	65.4 ± 10.6	21.8 ± 2.5	11.5 ± 4.7	6.0 ± 2.6

### Study Design, Tasks and Procedures

In the single visit, study participants performed different CoD tests, CMJs and FW squats for F-v assessment. Prior to the testing, they completed a 20-min warm-up consisting of 10-min of light running, performing arm, hip, knee and ankle mobility exercises (10 repetitions each), dynamic stretches of hip flexors, knee extensors, knee flexors and ankle extensors (10 repetitions each) and heel raise, squat, crunch resistance exercise (10 repetitions each). After that, participants performed CoD 90°, CoD 180°, *t*-test, and KST in random order. There was a 5-min break between each test to avoid the influence of fatigue. After CoD tests, the participants performed CMJs and FW squats with progressive loads in random order.

### CoD Testing

CoD tests were performed in a sports gym on a parquet, while participants wore indoor shoes. CoD tests and *t*-test were timed using photocell timing gates (Brower Timing Systems, Draper, UT, United States). In both cases, gates were placed at about hip height and 3 m apart. For each test, participants performed two familiarization trials at approximately 50 and 75% of their subjectively estimated maximal speed. The starting line was 0.5 m behind the first timing gate to prevent early triggering. For each side turn (preferred and non-preferred) and task (CoD 90°, CoD 180°) participants performed three maximal CoD trials in random order. There was a 1-min rest between consecutive trials and a 3-min rest between tasks (12 trials in total). During CoD execution, participants placed their preferred foot on the middle of the starting line. They started the test self-initiated and sprinted with maximal speed around the cone and made a 90° turn on one of the two sides and sprint through the finish line (second timing gate). The distance from the start timing gate to the CoD marking cone and the distance from the cone to the finish timing gate were both 5 m (total distance was 10 m). During the CoD 180° participants sprinted around the cone and back to the first timing gate, subsequently, the total distance was 10 m. The *t*-test was performed as suggested by Semenick, (1990). The participant started the test behind the starting point (starting line) on his own and sprinted forward 9.14 m to the first cone (touch with the right hand). The movement proceeded to the left using lateral movement and touched the left cone (4.57 m) with the left hand. The participant then shuffled to the right (9.14 m) and touched the right cone with the right hand. Afterward, the participant shuffled back to the middle cone (4.57 m, touch of the middle cone) and ran backwards to the finish line (time gate). Each participant performed three maximal trials with 3 min rest. The rest of the *t*-test was prolonged compared to CoD 180° to maintain the same work-to-rest ratio for each test. Although using only 1 min breaks is common for longer CoD tests (Raya et al., 2013), we chose to prolong the rest for *t*-test and not to reduce the rest for CoD 180° to minimize any potential effects of fatigue.

**FIGURE 1:**
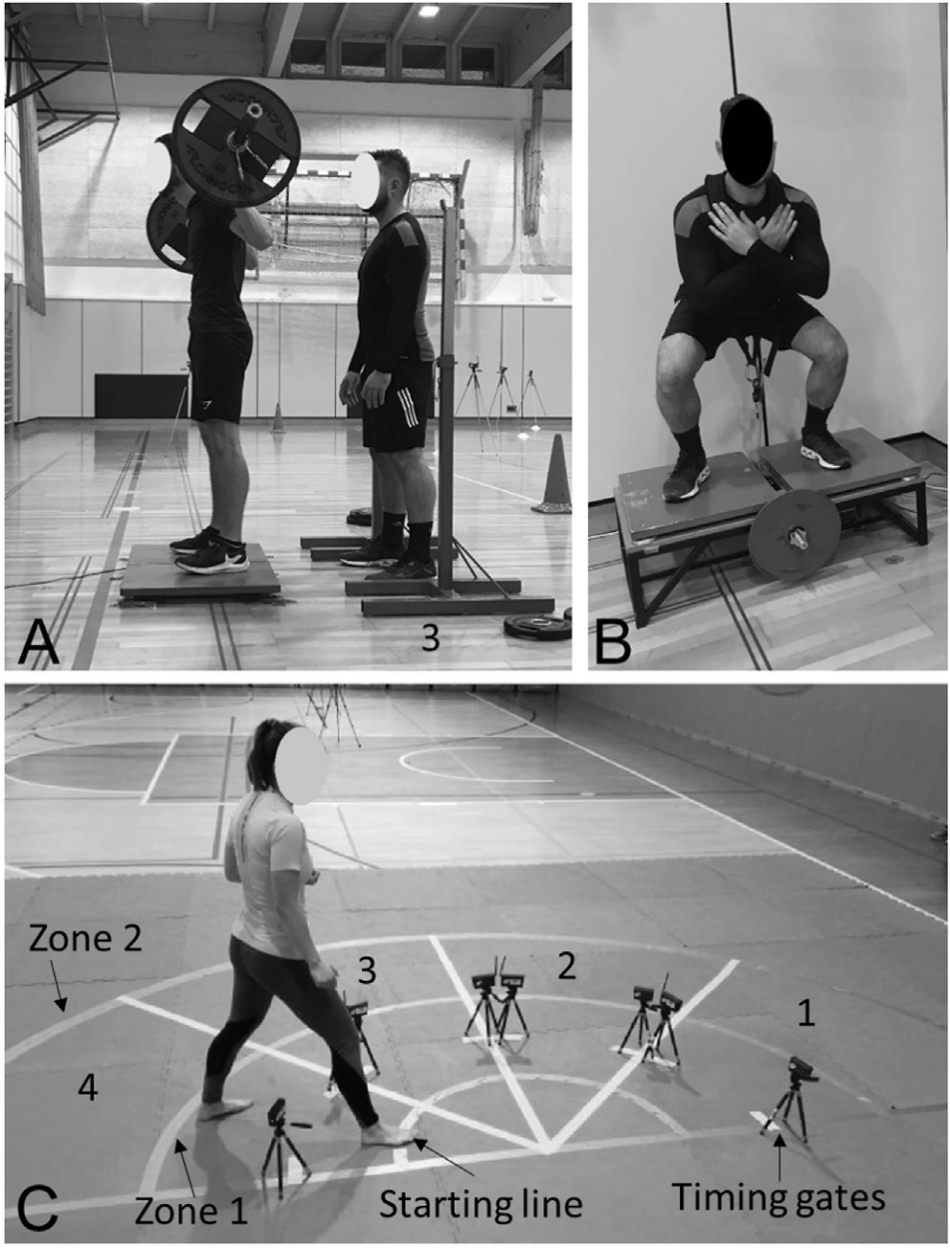
Measurement set-up for counter movement jumps **(A)**, flywheel squats **(B)** and karate specific test **(C)**.

The reliability of the KST test was confirmed in one of the previous studies (Smajla et al., 2021a). The test was performed on a tatami surface (Figures 1C). During the movement to the right participants were in the left guard (left leg in front) and the test started in section 1 (Figures 1C, sections are marked with numbers: 1, 2, 3, and 4). The front leg was placed behind the starting line while the back leg was placed freely behind. The photocell timing pair was placed between the front and the rear foot (Figure 1). The time started to run when the participant disrupted the timing gates with the front foot - moving backward in the left guard - with the intention to cross over the selected zone indicator with the front foot (Figure 1; zone 1, zone 2). Afterward, when their front foot was behind the zone indicator, they performed lateral movement to the right (to section 2) without placing the front foot across the selected zone After both their feet were in section 2 they performed forward movement in left guard to disrupt the timing gates by placing the front foot across the starting line. Immediately after backward movement was performed until the first foot was placed behind the selected zone line in section 2. Again, the lateral movement to the right was performed moving to section 3. Forward movement across the starting line and backward movement across the selected zone line was repeated in section three. Finally, the right lateral movement was performed to section 4 where participants performed the last forward movement across the blue line to finish the test. The same movement was performed in the right guard moving to the left side (from section 4 to section 1). Participants performed 3 trials in each direction (to the right in left guard, to the left in right guard). This protocol was performed in zone 1, where starting and zone line indicators were 1.5 m apart (short KST), and in zone 2 were this distance was 2.5 m (long KST). Altogether 12 trials were performed.

Loud verbal encouragement was provided for all CoD tests to ensure maximum effort. The main outcome measure was the best total time (s) for each test or condition within the test (left or right turn, left or right guard). Results of the CoD tests were organized regarding the leg which was responsible for the turn and participants self-reported preferred push-off leg (preferred, non-preferred). In the case of KST test results were organized based on participants’ self-reported preferred guard - based on their preferred front leg.

### Force-Velocity Profile During Countermovement Jump

Participants performed CMJs on a bilateral force plate (model 9260AA6, Kistler, Winthertur, Switzerland) with Kistler MARS software to record ground reaction force data. Each subject performed two to three submaximal introductory CMJs before the testing and one introductory repetition for each additional load. Test execution was supervised by the experienced researcher to improve proficiency in the jumping technique. Before each jump, participants were instructed to stand straight and still in the centre of the force plate. From this position, participants initiated a fast-downward movement until a crouching position with a knee angle of about 90°, followed by a jump for maximal height as quickly and explosively as possible. During the introductory trials, the appropriate depth was determined with a manual goniometer, and one of the examiners (positioned in a sagittal view) visually verified that the appropriate depth was used during all trials. At each load, three valid trials were performed with a 1-min recovery period (Petrigna et al., 2019). The rest between different loading conditions was set at 2 min. First, three CMJs were performed without an additional load. A lightweight (<0.5 kg) plastic bar was used instead of the barbell to ensure comparable hand position to the loaded conditions. This hand position remained the same during the entire movement. Afterwards, loaded jumps were performed starting with the 20 kg barbell. Then, the load was gradually increased by adding 10 kg until the athlete was able to jump about 10 cm high (Morin and Samozino, 2016). Average ground reaction force and take off vertical velocity data were measured for 3.9 (1.03) and 5.1 (0.8) additional loads for females and males, respectively. In total 4.5 (1.0) additional loads were used. The final load used in males was 60 kg (8 participants) and the final load in females was 50 kg (4 participants). Average ground reaction force produced during CMJ protocol corresponded to 64, 69, 73, 77, 80% an 85% of the CMJ-F_0_, from the body weight to the highest loading condition, respectively. Outcome measures entering the regression equation were manually checked and no loading condition was discarded due to deviation of the linear model. The testing protocol lasted approximately 15–20 min per participant, depending on the number of additional loads used. The jump with the maximal achieved height at each loading condition was taken for further analysis.

### Force-Velocity Profile During Flywheel Squats

Participants performed squats on a custom-made FW device used in previous studies (Spudić et al., 2020; Spudić et al., 2021a; Smajla et al., 2021b; Spudić et al., 2021b). Based on previous findings we used four FW loading conditions for all participants: 0.025, 0.075, 0.225 and 0.25 kgm^2^ (Spudić et al., 2020). While the absolute loads are not comparable between both F-v protocols, nor with other studies, both loading conditions were expressed relatively to F_0_ values. Selected loading conditions corresponded to the same region of F-v relationship (ie. moderate to high force or moderate to low velocity). Average ground reaction force produced during squats corresponded to 52, 65, 75, and 77% of the FW-F_0_ for 0.025, 0.075, 0.225 and 0.25 kg m^2^, respectively. To avoid a systematic inter-load effect, loads were administered in random order. A bilateral force plate system (type 9260AA, Kistler, Winterhur, Switzerland) was mounted on the FW device to record ground reaction forces. Simultaneously, vertical position data were acquired using a draw-wire sensor (Way-Con SX-50, Taufkirchen, Germany, range 1,250 mm, linearity ±0.02%). The sensor was mounted to the FW device, perpendicular to the FW shaft, below the standing surface. Sensor’s attachment site was at the lifting harness–between the legs. The two systems were synchronized with USB Data Acquisition System (Type 5695B, Kistler Instrumente AG, Winterhur, Switzerland). First, participants performed two sets of five to ten repetitions at a submaximal level with each load to adopt the correct technique and execution of the FW squat. Then, two sets of ten repetitions were performed for each loading condition. The first two repetitions were used to achieve correct squat execution (tempo and amplitude) and the last two to safely decelerate the spinning FW. For repetitions three through eight, participants were instructed to perform the concentric phase as quickly as possible and to delay deceleration during the first third of the eccentric phase and to make the transition from the eccentric to the concentric phase as short as possible. The execution of the squat was determined from the bottom position (approximately 90° knee angle) to the full extension of the knees (approximately 0° knee angle). To standardize the depth of the squat real-time vertical position feedback was displayed on the screen in front of the subject. Lifting the heels of the ground was not allowed, while hands were crossed at opposite shoulders. Rest periods between different FW loads were at least 2 min, allowing participants to maintain maximal power under the different FW loads (Sabido et al., 2020). Mean concentric velocity and ground reaction force variables were calculated as the average of six consecutive squat repetitions, as previously suggested (Spudić et al., 2020). The concentric part of the squat was defined as the amplitude from the lowest (approximately 90° knee flexion angle) to the highest squat amplitude (approximately 0° knee flexion angle) from the draw-wire sensor position data. The set with a higher mean velocity at each loading condition was taken for further analysis.

### Data Processing and Outcome Measures

Ground reaction force data during CMJ were sampled at 1,000 Hz, filtered using a moving average filter with 50-ms window and analysed using the Kistler MARS software built-in module for CMJ. The position data from the linear encoder and the ground reaction force data from the force plates during testing were acquired simultaneously at a frequency of 1,000 Hz and filtered using a 50 ms moving average filter (Harris et al., 2010; Spudić et al., 2020). For the purposes of this study, the average force and average velocity of the concentric part of the movement (CMJ jump and FW squat, respectively) were used. Based on force and velocity data at four FW loading conditions and incremental CMJs conditions, a least squares linear regression model (F [v] = F_0_-kv) was used to determine the four outcome measures of the F-v relationships, where F_0_ represents the force-intercept and k is the slope of the F-v relationship. The use of Equations 1 and 2 allowed for the calculation of V_0_ (maximal unloaded velocity, i.e., x-intercept) and P_max_: V_0_ = F_0_/k (1) and P_max_ = (F_0_∙V_0_)/2) (Samozino et al., 2012). Median (lower and upper 95% confidence interval for median) linear regression goodness of fit coefficient (R^2^) corresponded to 0,96 (0,94-0,99) in FW squats and 0,86 (0,79-0,90) for CMJs.

### Statistical Analysis

All statistical analyses were performed using SPSS (IBM SPSS version 26.0, Chichago, IL, United States) software package. Descriptive statistics of the dependent variables are presented as means and standard deviations. Normal distribution of data was assessed using Shapiro-Wilk tests, while homogeneity was assessed with Leven’s tests. The associations between CoD tests, training years and KST performance were assessed by Pearson’s correlation coefficients. The coefficients were calculated among F-v outcome measures assessed during CMJs and FW squats, as well as among F-v outcome measures (CMJ and FW) and CoD tests. Correlation coefficients were interpreted according to Hopkins et al. (2009) (0.00–0.19 trivial; 0.20–0.29 small; 0.30–0.49 moderate; 0.50–0.69 large; 0.70–0.89 very large; 0.90–0.99 nearly perfect; 1.00 perfect). Multiple linear stepwise regressions were done with all CoD tests (CoD 90°, CoD 180° and KST test) as individual dependent variables, while F-v outcome variables of CMJs and FW squats (F_0_, V_0_, P_max,_ slope) were included as candidate predictors. Durbin–Watson statistics and collinearity tests were performed. We conservatively set the thresholds for the presence of collinearity at ≤0.3 for tolerance and ≥3 for variance inflation factor. Additionally, visual inspection of a scatterplot of residuals was done to confirm homoscedasticity of the residuals. Paired samples *t*-test was used to identify absolute differences between the F-v outcome measures derived from FW squats and CMJs. Cohen’s d effect size (d) was used to quantify the magnitude of the differences, using the following interpretation: negligible (<0.2), small (0.2–0.5), moderate (0.5–0.8) and large (>0.8) (Cohen, 1988). Significance level was set at *p* < 0.05 (two-tailed).

## Results

The descriptive statistics and comparison of F-v calculated from CMJs and FW squats are presented in Table 2. Significant differences were calculated between all outcome measures.

**TABLE 2:** Descriptive statistics of force-velocity outcome variables calculated from countermovement jumps and flywheel squats.

Outcome measures	CMJs	FW squats	p (ES)
F_0_ (Nkg^−1^)	29.9 ± 4.3	27.0 ± 4.3	0.001 (0.60)
V_0_ (ms^−1^)	4.7 ± 1.5	2.0 ± 0.4	0.000 (1.84)
P_max_ (Wkg^−1^)	34.5 ± 7.4	13.3 ± 2.1	0.000 (3.02)
F-v_slope_ (Ns^−1^mkg^−1^)	−6.9 ± 2.3	−14.3 ± 4.7	0.000 (1.55)

F_0_, theoretical maximal force; V_0_, maximal unloaded velocity; P, theoretical maximal power; F-v slope, regression line of force-velocity relationship.

Analysis of relationship between F-v outcome variables assessed during CMJs and FW squats showed significant positive moderate correlations between CMJ-F_0_ and FW-F_0_ (r = 0.378, *p* < 0.018), and moreover, between CMJ-P_max_ and FW-P_max_ (r = 0.323, *p* < 0.045). There were no significant correlations between F-v outcome variables of both tasks regarding V_0_ (r = 0.132, *p* = 0.43) and slope (r = 0.226, *p* = 0.17).

Correlations between CoD tests and F-v outcome variables are presented in Table 3. Few significant correlations were found between CoD tests and F-v outcome variables calculated from CMJs, while only one significant correlation was found between CoD tests and F-v outcome variables calculated from FW squats (Table 3).

**TABLE 3:** Correlation between CoD tests and Force-velocity outcome variables calculated from countermovement jumps and flywheel squats.

	F_0_		V_0_		P_max_		F-v slope	
	CMJ	FW	CMJ	FW	CMJ	FW	CMJ	FW
CoD 90°	−0.39*	−0.09	0.22	−0.15	−0.01	−0.30	0.21	−0.09
CoD 180°	−0.58**	−0.22	0.37*	0.03	0.08	−0.21	0.43**	0.07
short KST	−0.12	−0.15	−0.06	−0.18	−0.22	−0.37*	−0.06	−0.04
long KST	−0.25	0.02	0.05	−0.20	−0.12	−0.23	0.12	−0.13
*t*-test	−0.53**	−0.10	0.32*	0.08	0.04	−0.21	0.35*	−0.02

CoD, change of direction test; CoD 90°, CoD test at 90°; CoD 180°, CoD test at 180°; KST, karate specific test; *p < 0.05; **p < 0.01.

In all regression models, only one predictor was included. Percentage of explained variance for each CoD test is presented in Table 4. The VIF and tolerance values for all regression analyses indicated that there was no multicollinearity present.

**TABLE 4:** Predictors and percentage of explained variance for change of direction tests.

CoD test	Predictor	R^2^
CoD 90°	CMJ-F_0_	0.154*
CoD 180°	CMJ-F_0_	0.355**
*t*-test	CMJ-F_0_	0.277**
short KST	FW-P_max_	0.138*

CoD, change of direction test; CoD 90°, CoD test at 90°; CoD 180°, CoD test at 180°; KST, karate specific CoD test; CMJ-F_0_, theoretical maximal force during counter movement jump; FW-P_max_, theoretical maximal power during flywheel squats.

## Discussion

This study was designed to compare F-v profiles measured during CMJs and FW squats, as well as to determine the correlations of F-v outcome measures (F_0_, V_0_, P_max_ and F-v_slope_) with different CoD performance variables (CoD 90°, CoD 180°, *t*-test and KST). Our main findings revealed significantly higher values of all F-v outcome variables calculated from CMJs compared to F-v outcome variables calculated from FW squats. Significant positive moderate correlations between the outcome parameters in CMJs and FW squats were found for F_0_ and P_max_, while no significant correlations were found in the case of V_0_ and F-v_slope_. In comparison to F-v outcomes obtained in FW squats, higher correlations were found between F-v outcome measures calculated from CMJs and CoD tests. The only significant correlation in F-v outcome measures calculated from FW squats was found between P_max_ and short KST time. For all CoD tests, only one F-v predictor was included; more specifically—CMJ-F_0_ for CoD 90°, CoD 180° and *t*-test, and FW-P_max_ for short KST performance.

To our knowledge, this is the first study that has reported the F-v profiles of elite karatekas and the first study that has compared F-v profiles during CMJs and FW squats. The comparison revealed significant differences in all F-v outcome variables calculated from each task. Significantly higher F_0_, V_0_, P_max_ and F-v_slope_ were calculated from CMJs compared to FW squats. The greatest absolute difference was observed in P_max_ as a consequence of lower values of V_0_ during FW squats, while F_0_ values differed less. The only moderate positive correlations between analogues outcome measures from CMJs and FW squats were found for F_0_ and P_max_ outcome variables (r = 0.323–0.378, *p* = 0.018–0.045). Based on the results we can partly confirm our first hypothesis. It is clear that in order to prevent the push-off in FW squats, an athlete must accelerate rapidly from the bottom of the squat and then reduce the acceleration before full leg extension. Lifting the heels of the ground during squats was not allowed to provide greater reliability of the mechanical variables (Spudić et al., 2020) but it could negatively influence mechanical output, especially the velocity of the movement. In contrast, the full leg extension is desirable in CMJ to accelerate body mass and additional load at a maximal distance, which results in the maximal push off velocity, and consequently, jump height. These results opened some questions regarding the direct comparison of the F-v profiles due to the difference in the tempo of the motor task execution. Future studies should be focusing on the determination of the optimal methodology for comparing the F-v outcomes between CMJ and FW squats (Spudić et al., 2021a). Moreover, it was shown that previous experience in the use of FW devices influences mechanical squat performance (Galiano et al., 2021; de Keijzer et al., 2022). Due to the specificity of performing the FW squat, the tempo execution lack of familiarization session in our study may have negatively affected the absolute force and velocity results, although the participants were elite karatekas with high training frequency and experience in strength resistance training experience. Nonetheless, during the testing protocol, maximal squat execution was ensured by clear instructions and loud verbal encouragement during the squats. FW resistance training has gained attention only in recent years and therefore the equipment is not frequently used, especially among karatekas. On the contrary, karatekas were all familiar with performing resistance exercises using a barbell. Therefore, it can be speculated that the results obtained from loaded CMJs are more trustworthy, and therefore, it might not be surprising that they correlate to a higher extent with the CoD tests. By means of an FW device, the method allows for significantly increased eccentric force demands compared to traditional resistance exercises (Norrbrand et al., 2011; Smajla et al., 2021b). Further, when performing an FW squat with delaying the braking action in the first third of the eccentric phase, greater eccentric than concentric peak force production can be achieved, which is known as an eccentric overload (Tesch et al., 2017; Spudić et al., 2020; Spudić et al., 2021a). It was previously found that, despite the full voluntary effort, neuromuscular activation of the quadriceps femoris muscle appears inhibited during slow and fast eccentric contractions (Aagaard et al., 2000) due to tension-limiting mechanism specific to eccentric action (Amiridis et al., 1996; Alcazar et al., 2019). High eccentric force demands in FW squats could have caused neural inhibition and therefore emphasized the differences between the FW and CMJ F-v profiles outcomes. Thus, the existence of a neural regulatory mechanism that limits the recruitment and/or discharge of motor units during maximal voluntary eccentric quadriceps contraction could have negatively influenced the average force produced in the concentric phase. In detail, lowering the muscle force in the eccentric part of the squat and simultaneously lowering the stretch of the tendons is transferred to the initiation of the upward movement. The lower ground reaction forces and lower forces acting on the muscle-tendon unit during the initiation of the upward movement do not allow a quick force transmission (Finni and Komi, 2000) moreover, the time for maximal active state development of cross-bridge formation is reduced (Arakawa et al., 2010) and therefore force and velocity production in the limited push-off time interval of the FW squat is lower (Van Hooren and Zolotarjova, 2017). Altogether, the concentric phase of the squat could be started from the favorable muscle activation state, initial force production and large elastic energy storage if the inhibitory effect is reduced. While the reduction of eccentric strength due to inhibitory mechanisms was found to be higher in sedentary subjects in comparison to strength athletes (Amiridis and et al., 1996), suggesting that the underlying mechanisms may be modulated by training, it could be speculated that a tempo of FW squats execution and a lack of experience in the eccentric overload training, in comparison to the barbell CMJ, increased the differences between the F-v outcomes. On the contrary, in strength trained athletes eccentric overload prior to the concentric phase of the front squat enhances the velocity and power of the vertical movement (Mugner et al., 2017). To summarize, the eccentric overloading during the FW squat may have induced superior, or at least similar levels of force, neural drive and loading on the muscle-tendon unit than CMJs. Thus, it could be hypothesized that eccentric overload might have also resulted in post-activation potentiation but to the author’s opinion, this advantage is not enough to compensate for the negative effects of precluding the push-off.

While optimal muscle-force sequencing during push-off affect ground reaction force application and jumping performance (Pandy and Zajac, 1991), the differences in the F-v outcomes could also be a consequence of a difference in the timing of muscle activation between loaded jumps and FW squats. While the lowest FW load follows the proximal-to-distal principle of muscle activation, higher FW loads require a specific and stable muscle coordination pattern, which is not proximal-to-distal (Spudić et al., 2021b). These findings are not in the line with (Giroux et al., 2015; van den Tillaar et al., 2019) which suggest that muscle coordination is not influenced by the external load during a ballistic squat jump and squats performed with maximal movement velocity, respectively. It could be speculated that the differences occur due to use of the harness in FW squats and barbell in loaded CMJ. Harness sits across the shoulders, chest, and lower back, evenly stressing the muscles crossing the hip joint and the spine erectors and could influence movement dynamics in the transition from the eccentric to the concentric part of the squat differently that a barbell.

Additional differences regarding the F-v outcome variables between CMJ jumps and FW squats could be a consequence of certain specific in terms of measurement protocols and data analysis. An average concentric phase force and velocity values of the highest jump at each loading condition were included in the F-v regression analysis. On the contrary, in FW squats, six consecutive repetitions were averaged to get trustworthy results (Spudić et al., 2020), which could have limited the insight into the maximal capacity for the execution of the FW task. From a practical point of view, our results show that stronger karatekas perform better on CoD 90°, CoD 180°, and *t*-test. More powerful karatekas in FW conditions show better performance in short KST. It appears that strength training is a key determinant of CoD ability. Specific lower extremity agility a in karate, as measured by short KST, has been shown to depend on power production during the FW squat. Therefore power-oriented training is desirable - regardless of the contribution of force and velocity to the common power production.

In the study performed by Jiménez-Reyes et al. (2018) karatekas showed greater F_0_ (33.8 0 ± 3.8 Nkg^−1^), lower V_0_ (3.0 ± 0.3 ms^−1^) and P_max_ (25.5 ± 3.8 W kg^-1^), however, these results cannot be directly compared with ours because they performed squat jumps. It is known that F-v relationships during CMJ have larger F_0_, V_0_ and consequently P_max_ compared to F-v relationships during SJ since mechanical characteristics of eccentric-concentric movements (CMJs) are superior to that registered in purely concentric actions (SJ) (Jiménez-Reyes et al., 2014). Moreover, the characteristics of the karatekas in the aforementioned study were not specified in detail, as they were a part of a larger sample but we can assume that the karatekas sample of our study was weaker than the sample of Jiménez-Reyes et al. (2018). Compared to sprinters and jumpers (F_0_: 38.0 ± 4.9 Nkg^−1^, V_0_: 4.6 ± 0.8 ms^−1^, P_max_: 42.9 ± 5.6 W kg^-1^, F-v_slope_: 8.8 ± 2.8 Ns^−1^mkg^−1^) our participants generally showed lower values (Jiménez-Reyes et al., 2014). This could be explained by the difference in the lower age (Rodríguez-Rosell et al., 2017) of our participants (karatekas: 19.3 ± 4.1 years, sprinters and jumpers: 23.1 ± 4.4 years) and the differences in training regimes, even though F-v_slope_ of both groups (sprinters and jumpers) show that their training history made them develop velocity capacity more than force capacity (Jiménez-Reyes et al., 2014, 2019; Cuk et al., 2016). On the other hand, there is a lack of evidence about F-v profiles measured during FW squats in other sports. Further studies are needed to support the assumption that karatekas’ power production abilities during FW squats depend on high velocity rather than high force production. Compared to physically active participants, experienced in resistance training (F_0_: 30.8 ± 6.9 Nkg^−1^, V_0_: 1.62 ± 0.3 ms^−1^, P_max_: 12.5 ± 3.5 W kg^-1^, F-v_slope_: 18.9 ± 5.8 Ns^−1^mkg^−1^) (Spudić et al., 2020), our participants showed slightly higher values. To the best of knowledge, there are no additional studies reporting F-v profiles obtained from FW squats. The results can be also influenced by specific and constantly repeated movements in martial arts. Namely, one of the studies showed that high level martial arts athletes have less eccentric loading and greater power production in the concentric phase (James et al., 2020). We can speculate that performing CMJ compared to FW squat represents a more similar movement that occurs during specific karate actions during which fast force production and fast preparatory countermovement is beneficial for their performance. On the other hand, strong eccentric deceleration such as in FW squats is rarely present in karate actions.

Our previous indications were confirmed with the higher correlations between F-v outcome measures and CoD tests for CMJs. Based on that we confirmed our second hypothesis. However, none of the CMJ F-v outcome variables were in correlation with KST. CMJ-F_0_ was in moderate to large negative correlation with both CoD tests and *t*-test (from −0.39 to −0.58, *p* < 0.01), while CMJ-V_0_ and CMJ-F-v_slope_ were in positive moderate correlation with CoD 180° and *t*-test (r = 0.32–0.43, *p* < 0.05). Participants with higher CMJ-F_0_ and higher CMJ-V_0_ outperformed other participants in CoD and *t*-test. Baena-Raya et al. (2020) reported a small negative association between 505 CoD and CMJ-F_0_ in soccer, tennis and basketball players (r = −0.245 to −0.279), while a moderate correlation was seen for CMJ-P_max_ (r = −0.378). Surprisingly, we found no statistically significant correlations between CMJ-P_max_ and CoD performance. Previous research (Young, 2006) on neural adaptations to resistance training indicates that intermuscular coordination is an important component in achieving transfer to sports skills and it could be speculated that CMJ and CoD tests cover different movement patterns from a coordination point of view. Moreover, Baena-Raya et al. (2021d) found stronger associations of the horizontal F-v profile (sprints) with COD performance compared to the vertical F-v profile (CMJ). Considering the fact that movement in KST is performed mostly in horizontal directions we may also expect greater associations with horizontal F-v profile.

This is the first study that investigated correlations of F-v outcome variables calculated from FW squats with CoD ability in karatekas. High power and force production in sport-specific situations maximizes the effectiveness of the movement. In general, we found distinguished associations between F-v outcome measures and CoD tasks. Our results showed that high force production (CMJ–F_0_) is associated with performance in running CoD performance, while power production (P_max_) during FW squats was an important factor for performance in the short KST. Moreover, adaptations of muscle structure, CoD, squat jump, sprint performance, and hamstring/quadriceps strength ratio have been reported to be specific to the load and velocity of movement (Coratella et al., 2018). Based on these findings, we can assume that velocity capabilities among karatekas are emphasized due to sport-specific demands and the training history (i.e., predominantly based on bodyweight exercise without additional external resistance). The only significant negative correlation regarding FW squats F-v outcome variables was seen between P_max_ and short KST (r = −0.37, *p* < 0.05). This result suggests that more powerful karatekas could be faster during KST performance. The results are in line with the results of Tous-Fajardo et al. (2016) who had shown that improved power after FW resistance training positively affects CoD performance in soccer players. During the short KST, karatekas perform multiple CoD on short distances (0.5 m), stressing deceleration and acceleration ability. Such movements require higher eccentric strength, which is also needed in FW squats. This was also supported in our regression analysis as P_max_ calculated from FW squat was the only predictor of short KST test, with 13.8% of explained variance. Previous studies found a moderate negative association between maximal power produced during FW squats and CoD (Suarez-Arrones et al., 2020). However, in their case, the best mean power was calculated during FW squats with 0.10 kgm^2^ load and not F-v profile—therefore a direct comparison of the results is not trustworthy. Nevertheless, only a small part of the variance was explained by the F-v outcome variables. This could be a consequence of test selection. F-v profiling using average values of force and velocity during a concentric part of the squat does not reflect the rate of force development during a movement. The latter was shown to be a more specific outcome variable than peak or average force when describing sport-performance tasks in time-limited force production situations (Hernández-Davó and Sabido, 2014). This is especially relevant in karate, where CoD speed is more important than maximal force (Ioannides et al., 2020). Further on, the results can be correlated to the type of karate technique. Kicking and punching performance is highly dependent on the explosiveness of movement, i.e. rate of force development at the beginning of the movement and not the final speed of the movement—while direct full contact with the opponent is prohibited. Specific technique demands could have influenced karatekas neural and muscle strength adaptations, which were not measured using F-v profiling, and therefore the generalisability of the testing procedure to CoD tasks could be limited.

F_0_ during CMJs was the only predictor for the remaining CoD test apart from short KST. It explained 15.4% of variance for CoD 90°, 35.5% of the variance for CoD 180° and 13.8% of the variance for short KST. It is known that greater average braking and propulsions forces are present when CoD is performed at a sharper angle (Schreurs et al., 2017; Dos’Santos et al., 2018). During sharper cuts, the force component is more pronounced, while the velocity component is more important in lower angle cuts (Falch et al., 2020). This is also supported by our results, as F_0_ was a better predictor for the tests with greater CoD (CoD 180° and *t*-test). During kumite fights, most CoDs occur at angles <90°, therefore it is not surprising that mean values of F-v_slope_ in our case indicate karatekas velocity dominance. Based on our results we can reject our third hypothesis.

There are several limitations with the testing procedure that should be noted. Familiarization to FW squats in our study was shorter than suggested (Sabido et al., 2018). Moreover, the exclusion of the eccentric part of the squat is one of the limitations of the study. We strongly believe that force and velocity variables in the eccentric portion of the squat should be analyzed in the future, while training with accentuated eccentric muscle actions was shown to be a promising element in strength and conditioning programs of sports with high CoD speed demands (Chaabene et al., 2018). One of the reasons why the eccentric part of the squat was excluded from the analysis was a lack of studies in the literature on the reliability of the eccentric F-v profile, especially for FW squats. In comparison to concentric contraction, the eccentric muscle contraction follows specific neural and mechanical demands and we believe that reliability and validity of eccentric F-v profiling should be considered first, and then the results of the reliable eccentric protocol should be compared between different measures - to check concurrent validity. Finally, a relatively long distance is covered in *t*-test, which makes it non-specific to karate. Using a modified *t*-test would perhaps be more appropriate for this study.

## Conclusion

In conclusion, our results showed significant differences in F-v outcome variables calculated from CMJs and FW squats due to different demands in both tasks. Moreover, we confirmed that high force and power production is important for the successful performance of general and karate-specific CoD tasks In general, the CMJ-F-v profile provides additional information about the karateka’s agility performance, making its use in karate preferable to using FW-based profiling. The only exception is the short KST, for which the FW -P_max_ profile is the most informative measure. Despite that FW devices are easy to use for training (they are portable, the load is easy to adjust, and the use of harness unloads lower back), sports practitioners and researchers should be aware of the discrepancy in practical information between the FW and CMJ protocols, interpret the results carefully, and use each one according to their interest (short KST vs. other agility abilities in karatekas). Future interventional studies are needed to determine the optimal training recommendations for this purpose. In the future, the results from the F-v profiles and CoD test should be also correlated to the rate of success in karate.

## Data Availability

The raw data supporting the conclusions of this article will be made available by the authors, without undue reservation.
